# Clinical outcomes following a hospital-wide transition from enoxaparin to tinzaparin for venous thromboembolism prophylaxis in neurosurgical patients

**DOI:** 10.1007/s00701-026-07027-7

**Published:** 2026-09-25

**Authors:** Leonard Ritter, Adrian Liebert, Jens Weigel, Karl M. Schebesch, Thomas Eibl

**Affiliations:** Department of Neurosurgery, Paracelsus Medical University, Nuremberg General Hospital, Breslauer Straße 201, 90471 Nuremberg, Germany

**Keywords:** Thromboembolism, Neurosurgery, Anticoagulation, Efficacy, Tinzaparin, Enoxaparin, Surgery

## Abstract

**Objective:**

Venous thromboembolism (VTE) prophylaxis is a key component of neurosurgical care, with low-molecular-weight heparins (LMWHs) representing the standard pharmacological approach. While enoxaparin is well established, the evidence regarding the efficacy and safety of tinzaparin in neurosurgical patients remains limited. This study aimed to compare the incidence of VTE and postoperative rebleeding between enoxaparin and tinzaparin in a large cohort of neurosurgical patients.

**Methods:**

Following a standardized institutional switch from enoxaparin to tinzaparin in 2021, we conducted a retrospective cohort study including all adult patients undergoing neurosurgical procedures during the 2.5-year periods before and after the change. Patients were stratified according to their perioperative VTE risk. The primary outcome was imaging-confirmed symptomatic VTE during hospitalization, and the secondary outcome was postoperative rebleeding requiring surgical evacuation. A multivariable logistic regression analysis was performed to adjust for potential confounders.

**Results:**

A total of 4,888 patients were included. Overall, 51 symptomatic VTE events occurred (1.04%). In low risk patients (Group A), VTE incidence did not differ significantly between enoxaparin and tinzaparin (0.44% vs. 0.86%, *p* = 0.35). In moderate-to-high risk patients (Group B), VTE occurred significantly more frequently with tinzaparin than enoxaparin (1.67% vs. 0.84%, *p* = 0.04), driven predominantly by PE (1.05% vs. 0.18%, *p* = 0.01), while DVT rates were comparable (0.62% vs. 0.67%, *p* = 0.87). Multivariable analysis in Group B confirmed lower odds of VTE with enoxaparin (OR 0.475, 95% CI 0.246–0.919, *p* = 0.027). Postoperative rebleeding rates did not differ significantly between treatment groups. The increase in PE coincided with increased CTA utilization and showed no clear temporal step change following the switch to tinzaparin.

**Conclusion:**

Both enoxaparin and tinzaparin were associated with low VTE rates and comparable postoperative rebleeding rates. Although higher VTE rates with tinzaparin were observed in the medium-to-high risk group, the predominance of PE, increased CTA utilization, and absence of a temporal step change limit a causal interpretation. These findings should therefore be considered hypothesis-generating rather than evidence of superior efficacy of either agent.

## Introduction

Venous thromboembolism (VTE) prophylaxis is a critical component of the overall treatment concept in surgery, with low molecular weight heparins (LMWHs) serving as the standard thromboprophylactic agents across most surgical disciplines [17, 25]. Currently, various LMWHs are available for use in surgical settings, including ardeparin, dalteparin, enoxaparin, nadroparin, reviparin, and tinzaparin [16].

Currently, only few studies in the literature have directly compared different LMWHs in surgical patients. Most research to date has focused on comparisons between LMWHs and other anticoagulants, such as standard heparin or warfarin. The existing systematic reviews and meta-analyses comparing different LMWHs focus mainly on orthopaedic and general surgical patients. This circumstance is unfortunate as the prophylaxis in neurosurgical patients plays an especially important role within the surgical disciplines due to the particularly high incidence rates of thromboembolism of up to 50% without prophylaxis [11, 13].

Many of the neurosurgical patients are particularly prone to developing thromboembolisms due to factors such as prolonged surgery times, extended hospital stays, and hormonally altered coagulation status. For instance, Fischer et al. reported a threefold higher incidence of thromboembolism in neurosurgical patients compared to orthopaedic patients (1.87% vs. 0.61%) in a study involving 7,156 patients who underwent spinal surgery [8].

In 2021, our center implemented tinzaparin as the standard agent for VTE prophylaxis, replacing enoxaparin. Since then, all neurosurgical patients have received tinzaparin for in-hospital thromboembolism prophylaxis. A retrospective analysis was now conducted to compare the effectiveness of tinzaparin and enoxaparin in preventing in-hospital thromboembolic events. Additionally, the safety profiles of both agents were evaluated by analyzing the incidence of postoperative rebleedings requiring surgical revision.

To the best of our knowledge, no prior study has specifically compared the efficacy and safety of enoxaparin and tinzaparin in a purely neurosurgical patient population.

## Methods

### Ethical standard

The study was conducted according to the Declaration of Helsinki and its later amendments. No study-specific examinations were conducted. The study design was approved by our institutional review board (IRB-2024–13).

### Inclusion and exclusion criteria

This retrospective study included consecutive patients aged ≥ 18 years who underwent neurosurgical procedures between 2018 and 2023 and received pharmacological venous thromboembolism (VTE) prophylaxis with either tinzaparin or enoxaparin.

Patients undergoing ambulatory surgery or those discharged on the first postoperative day were excluded.

To ensure a representative cohort of the typical neurosurgical population, we included patients undergoing cranial, spinal, functional, and peripheral nerve procedures.

### Data collection and outcome measures

Demographic characteristics, medical history, intraoperative details, and imaging studies were retrospectively reviewed.

**Fig. 1:**
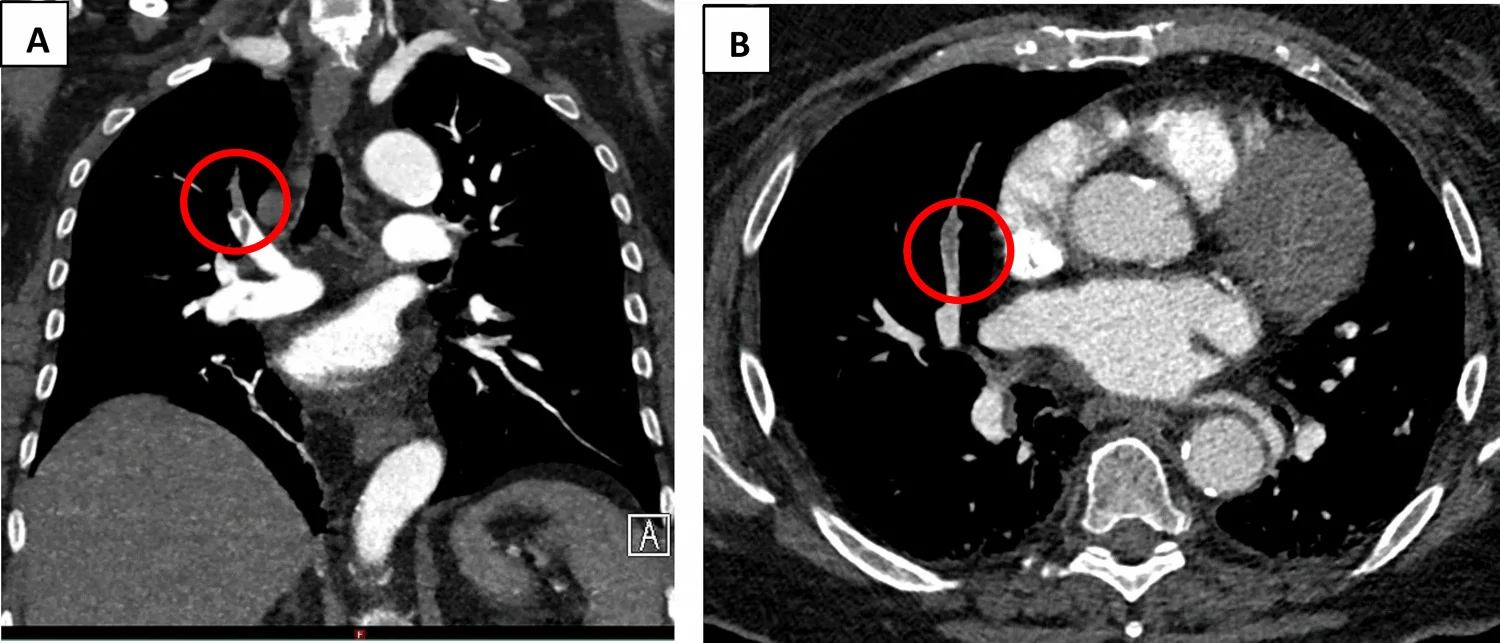
Computed tomography pulmonary angiogram (CTPA) with evidence of pulmonary embolism. Coronal (**A**) and axial (**B**) view of a CTPA with evidence of pulmonary emboli, visible as filling defect of contrast medium in a pulmonary segment artery on the right side

**Fig. 2:**
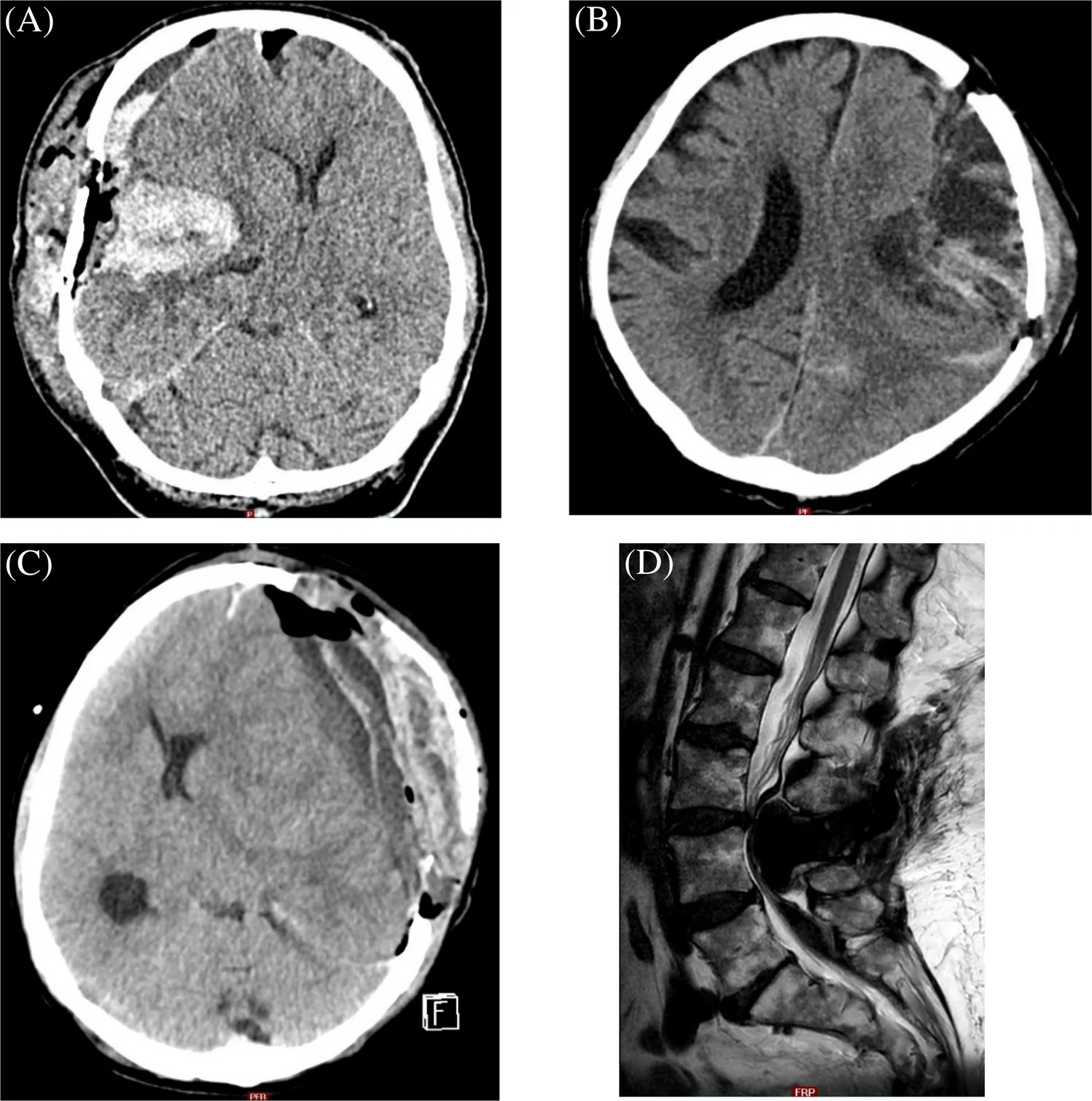
Rebleedings needing surgical revision. Intracerebral (**A**), epidural (**B**), subdural (**C**) and spinal epidural (**D**) rebleeding

The primary outcome was the in-hospital incidence of clinically apparent VTE, including deep vein thrombosis (DVT) and pulmonary embolism (PE), following neurosurgical intervention (Fig. 1). No routine screening with computed tomography angiography (CTA) or duplex ultrasonography was performed; only symptomatic events were recorded. No post-discharge follow-up was conducted, and events occurring after hospital discharge were not considered (Fig. 2).

The secondary outcome was the incidence of postoperative rebleedings requiring surgical hematoma evacuation. Rebleedings were categorized as spinal epidural, cranial epidural, subdural, or intracerebral hematomas.

Only events occurring from postoperative day two were included in the analysis.

### Diagnosis of pulmonary embolism and deep vein thrombosis

The diagnosis of pulmonary embolism (PE) and deep vein thrombosis (DVT) was based on established clinical guidelines and defined in this study as follows [14, 15, 21]:

- Pulmonary embolism was diagnosed only in patients who developed acute cardiorespiratory symptoms postoperatively. Confirmation required a CTA, demonstrating an intravascular filling defect consistent with PE.
- Deep vein thrombosis was diagnosed in the presence of acute clinical symptoms, including limb pain, swelling, or redness. Diagnosis was confirmed by a positive finding on Doppler ultrasound examination.

Only symptomatic cases confirmed by imaging were included in the analysis.

### Risk profiles for thromboembolic events

Within the neurosurgical patient population, there is a high degree of heterogeneity regarding the risk profile for VTE following a surgical procedure [4]. This means that conclusions regarding the efficacy of thrombosis prophylaxis for the entire neurosurgical patient population can only be drawn to a very limited extent. To increase the informative value of our study, we therefore divided the patients into two groups. Patients with a low risk were assigned to group A and patients with a moderate-to high-risk for thromboembolic events were assigned to group B.

Patients assigned to group B had a higher risk of VTE either due to their disease or the resulting surgery. Predictive factors in this group included prolonged LOS, immobility or bed rest, an altered coagulative status or a prolonged operation time [8, 12, 19].

The classification of patients into two groups also corresponded to current guidelines on risk profiles of different surgical procedures (Table 1) [18].

**Table 1:** Distribution of neurosurgical procedures according to their risk profile

Low risk (Group A)	Moderate-to-high risk (Group B)
Functional procedures Peripheral procedures Simple spine procedures	Intracranial procedures • Tumor • Aneurysmal subarachnoid haemorrhage • Infection • Spontaneous ICB Complex spine procedures • Instrumentation • Infection • Tumor Trauma Hydrocephalic procedures

### Thromboembolism prophylaxis treatment regime

Following the current guidelines, thromboembolism prophylaxis in our center was done with low molecular weight heparins [2, 4, 5].

Enoxaparin was administered at a daily dose of 4000 IU (international units), while tinzaparin was given at a daily dose of 3500 IU to patients at low risk and 4500 IU to patients at moderate-to-high risk for VTE. Tinzaparin dosing was performed according to institutional protocols based on the manufacturer's prescribing information and the national S3-guideline for venous thromboembolism prophylaxis of 2015.

Thromboprophylaxis was routinely initiated on the evening of the first postoperative day. In patients who underwent cranial procedures, prophylaxis was started only after postoperative imaging had been performed. The overall regime was the same for tinzaparin and enoxaparin and stayed unchanged during the whole duration of the study.

The aim was to mobilize all patients as early as possible. In cases where this was not possible, e.g. during a stay in the intensive care unit or during bed rest, physiotherapy and occupational therapy exercises were carried out in bed as far as possible.

### Statistical analysis

Statistical analyses were performed using IBM SPSS® Version 29 for Windows 10 (IBM Corp. Released 2020. IBM SPSS Statistics for Windows, Version 29.0. Armonk, NY: IBM Corp). Continuous variables are presented as medians with interquartile range (IQR). Categorical variables are reported as absolute numbers and percentages. Comparisons of continuous variables were conducted using the Mann–Whitney U test and Kruskal–Wallis test, while Fisher's exact test and Fisher-Freeman-Halton test were used for categorical variables. Subsequently, a multivariable analysis was performed to evaluate the robustness of the associations observed in the bivariate analysis of VTE events to adjust for potential confounders. The significance level was set to *p* < 0.05 in two-tailed testing.

## Results

### Patient population

A total of 4,888 patients were included in this study. Of them 2,570 patients received enoxaparin during period 1, whereas 2,318 patients received tinzaparin during period 2. 1,616 patients were classified as low risk (group A) while 3,272 patients had a medium-to-high risk (group B).

In group A (Table 2), 46.3% of patients in the enoxaparin group and 44.4% in the tinzaparin group were female, with a median age of 58 years in both groups. Spinal procedures were the most common type of surgery, accounting for 84.1% of procedures in the enoxaparin group and 75.1% in the tinzaparin group (*p* < 0.01). The median duration of surgery was significantly longer in patients receiving tinzaparin than in those receiving enoxaparin (117 vs. 95 min, *p* < 0.01). No significant differences were observed regarding length of hospital stay or duration of hospitalization before surgery.

**Table 2:** Low risk (group A)

Characteristics	Enoxaparin	Tinzaparin	*p*-value
Patient number	**913**	**703**	
Gender			
- Male	490 (53.7)	391 (55.6)	0.45
- Female	423 (46.3)	312 (44.4)	
Age	58 (25)	58 (25.5)	0.97
Category of surgery			
- Functional & peripheral procedures	138 (15.1)	175 (24.9)	< 0.01
-Spine procedures	775 (84.1)	528 (75.1)	
Duration of surgery (min)	95 (72.5)	117 (70.5)	< 0.01
LOS (days)	5 (3)	4 (3)	0.71
Duration of hospitalisation before surgery (days)	1 (2)	1 (3)	0.09
Events			
- DVT	3 (0.33)	3 (0.43)	0.75
- PE	1 (0.11)	3 (0.43)	0.3
- Total	4 (0.44)	6 (0.86)	0.35

*LOS* Length of hospital stay, *DVT* Deep vein thrombosis, *PE* Pulmonary embolism

In group B (Table 3), 44.8% of patients in the enoxaparin group and 45.2% in the tinzaparin group were female. The median age was 70 years in the enoxaparin group and 66 years in the tinzaparin group (*p* = 0.07). Cranial procedures were the most common type of surgery, followed by trauma, spinal, and hydrocephalus procedures. No significant differences were observed between the treatment groups regarding sex, age, surgical category, duration of surgery, or overall length of hospital stay. However, patients receiving tinzaparin had significantly longer preoperative hospitalization than those receiving enoxaparin (median 1 (4) vs. 1 (3) days, *p* < 0.001).

**Table 3:** Medium-to-high risk (group B)

Characteristics	Enoxaparin	Tinzaparin	*p*-value
**Patient population**	**1657**	**1615**	
Gender			
- Male	914 (55.2)	885 (54.8)	0.86
- Female	743 (44.8)	730 (45.2)	
Age	70 (26)	66 (24)	0.07
Category of surgery			
- Cranial procedures	682	728	0.48
- Trauma	437	412	
- Hydrocephalus	142	140	
- Spine	396	334	
Duration of surgery (min)	121 (140)	139 (162)	0.16
LOS (days)	8 (9)	7 (11)	0.79
Duration of hospitalisation before surgery (days)	1 (3)	1 (4)	< 0.001
Events			
- DVT	11 (0.67)	10 (0.62)	0.87
- PE	**3 (0.18)**	**17 (1.05)**	**0.01**
- Total	**14 (0.84)**	**27 (1.67)**	**0.04**

*LOS* Length of hospital stay, *DVT* Deep vein thrombosis, *PE* Pulmonary embolism

### Thromboembolic events

In group A, 4 VTE events occurred in patients receiving enoxaparin (3 DVTs and 1 PE) and 6 in patients receiving tinzaparin (3 DVTs and 3 PEs), with no significant difference in the overall incidence of VTE (0.44% vs. 0.86%, *p* = 0.35). In group B, 14 VTE events occurred in the enoxaparin group (11 DVTs and 3 PEs) and 27 in the tinzaparin group (10 DVTs and 17 PEs). While the incidence of DVT did not differ significantly between groups (0.67% vs. 0.62%, *p* = 0.87), pulmonary embolism occurred significantly more frequently in patients receiving tinzaparin (0.18% vs. 1.05%, *p* = 0.01). Overall, the incidence of VTE was significantly higher in the tinzaparin group (0.84% vs. 1.67%, *p* = 0.04).

Across the entire study population, the overall incidence of thromboembolic events was 1.04%.

### Multivariable analysis

As we observed a significantly higher incidence of pulmonary embolism among patients receiving tinzaparin in the medium-to-high-risk group, a multivariable logistic regression analysis was subsequently performed within this subgroup to account for potential confounding factors.

The dependent variable was the occurrence of symptomatic VTE (0 = no VTE; 1 = VTE), with a total of 41 VTE events. Tinzaparin served as the reference category. Based on the bivariate analysis, age and preoperative duration of hospitalization were included as covariates in the adjusted model.

Enoxaparin was associated with significantly lower odds of VTE compared with tinzaparin (OR 0.475, 95% CI 0.246–0.919, *p* = 0.027). Age was also significantly associated with VTE (OR 0.973, 95% CI 0.951–0.995, *p* = 0.017). whereas preoperative duration of hospitalization was not independently associated with VTE (OR 0.988, 95% CI 0.944–1.034, *p* = 0.596) (Tables 4 and 5).

**Table 4:** Result of the logistic regression analysis

Pharmacological agent	β	SE	*P*-value	Odds Ratio Exp(B)	95% CI
Tinzaparin 4500 IE	-	-	-	1.00	Reference
Enoxaparin	**- 0.744**	**0.336**	**0.027**	**0.475**	**0.246–0.919**
Age	- 0.028	0.012	0.017	0.973	0.951–0.995
Duration of hospitalisation before surgery	- 0.012	0.023	0.596	0.988	0.944–1.034

*P*-value was calculated by using logistic regression with age, duration of hospitalisation before surgery and pharmacological agent. *β* regression coefficient, *SE* standard error, *CI* Confidence interval

**Table 5:** Crude and adjusted odds ratios for venous thromboembolism in medium-to-high-risk patients (Group B)

Analysis	Odds ratio	95% CI	*P*-value
Crude/Unadjusted	0.5	0.25–1.00	0.04
Adjusted*	0.475	0.246–0.919	0.027

*Adjusted for age and duration of hospitalization before surgery. Tinzaparin served as the reference category. *CI* Confidence interval

While the crude odds ratio for VTE comparing enoxaparin with tinzaparin was 0.50, the association remained largely unchanged after the logistic regression analysis with an odds ratio of 0.475 (95% CI, 0.246–0.919; *p* = 0.027). Thus, adjustment for the included covariates had only a limited effect on the estimated association.

### Temporal analysis of VTE events

To further contextualize and interpret the findings of the bivariate and multivariate analyses, two additional aspects were examined in greater detail: the temporal overlap of the study period with the COVID-19 pandemic and the marked increase in pulmonary embolisms despite a relatively stable incidence of deep vein thrombosis.

**Fig. 3:**
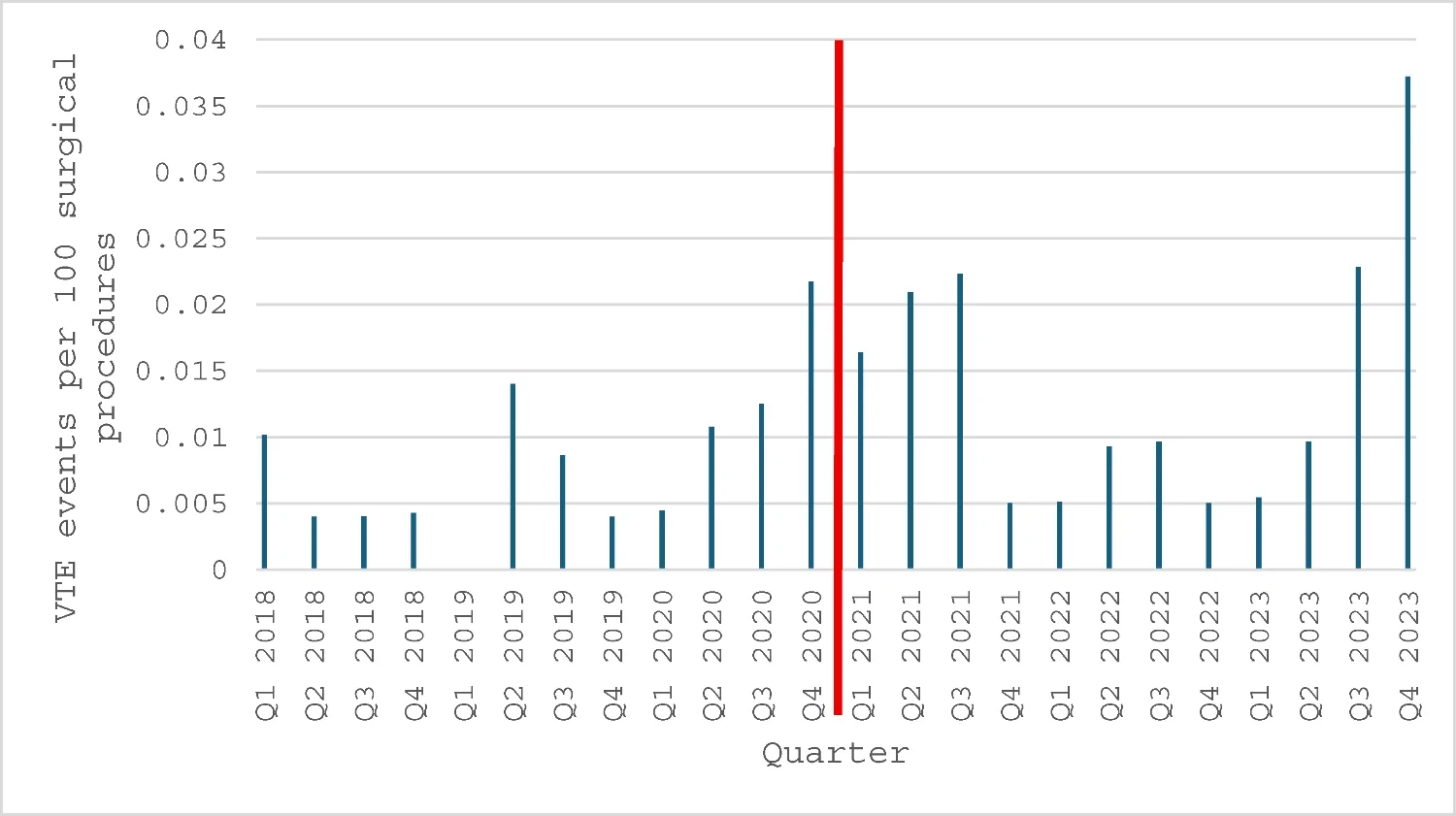
Plotted VTE events over the whole study period. The vertical red line marking the transition from enoxaparin to tinzaparin

All VTE events were plotted chronologically to assess their temporal distribution and to determine whether the increase in events coincided with the transition to tinzaparin (Fig. 3). The temporal distribution showed a predominantly episodic pattern of VTE events throughout the study period rather than a distinct and sustained step change following the transition to tinzaparin.

The frequency of CTAs was assessed to evaluate whether the threshold for performing CTA may have changed between the two study periods. In period 1, 164 CTAs were performed, corresponding to 0.064 CTAs per patient. In period 2, 222 CTAs were performed, corresponding to 0.096 CTAs per patient, representing an approximately 50% increase in CTA. The diagnostic yield of CTA also increased between the two periods, with positive findings in 2.4% of examinations in period 1 compared with 9.0% in period 2.

### Incidence of rebleedings

In the second part of the study, we compared the incidences of postoperative rebleedings between patients treated with tinzaparin and those treated with enoxaparin. Overall, the rates of rebleedings were low and statistically comparable between groups (1.36% vs. 1.42%, *p* = 0.9) (Table 6). No significant differences were observed in the occurrence of specific hematoma types, including spinal epidural, cranial epidural, subdural, or intracerebral hematomas.

**Table 6:** Incidence of rebleedings in tinzaparin vs. enoxaparin treated patients

Event	Enoxaparin	Tinzaparin	*P*—Value
Overall	35 (1.36%)	33 (1.42%)	0.9
Spinal epidural hematoma	11	12	0.68
Epidural hematoma	6	8	0.59
Subdural hematoma	6	5	1.0
Intracerebral hematoma	12	8	0.66

## Discussion

Our study aimed to compare clinical outcomes following a hospital-wide transition from Enoxaparin to Tinzaparin for VTE prophylaxis in neurosurgical patients.

Numerous in vivo and in vitro coagulation tests have demonstrated measurable pharmacodynamic differences between LMWHs when administered at comparable anti-FXa doses [23]. Additionally, tinzaparin and enoxaparin differ in their production methods—tinzaparin is derived through heparinase digestion, while enoxaparin undergoes benzylation followed by alkaline hydrolysis. These differences extend to their average molecular weights (5,000 daltons for tinzaparin vs. 4,300 daltons for enoxaparin) and half-lives (3 h for tinzaparin vs. 4.5 h for enoxaparin) [6, 7, 10]. As a result, these pharmacological distinctions may influence their effectiveness in thrombosis prophylaxis.

### Comparison of VTE events

In our study, the incidence of thromboembolic events was 0.62% in the low-risk group and 1.25% in the medium-to-high-risk group, resulting in an overall incidence of 1.04% for the whole patient population. Reported rates of VTE despite chemical thromboprophylaxis following neurosurgery or comparable surgical procedures vary significantly in the literature. Some studies report similar incidences, such as Fischer et al., who found a rate of 0.94%, whereas others, like Wilhelmy et al., report significantly higher rates (e.g., 4.3%) [8, 24]. These discrepancies arise due to differences in defining thromboembolic events, the heterogeneity of study populations, the length of the follow-up interval and variations in diagnostic methods. VTEs may be detected through autopsy, iodine-125 (^125I)-labeled fibrinogen studies, Doppler ultrasound, or CTA in symptomatic patients [1, 9]. Given these confounding factors, direct comparisons between studies are inherently limited.

While in the low-risk population no significant difference in VTE incidence was observed between enoxaparin and tinzaparin, there was a significant difference in the medium-to-high-risk population. Notably, this difference was driven by PE, with a significantly higher incidence in patients receiving tinzaparin, whereas DVT incidence was comparable between groups.

Based on these findings, a multivariable logistic regression analysis was performed within this subgroup. Here, enoxaparin remained associated with lower odds of VTE compared with tinzaparin. The crude estimate was highly similar, indicating that adjustment for the included covariates had little effect on the observed association.

To further contextualize these findings, additional analyses were performed to investigate potential temporal and diagnostic confounders. Three observations in particular question whether the observed association represents a true difference in pharmacological efficacy. First, the increase in VTE was almost exclusively attributable to PE, while DVT incidence remained virtually unchanged. This discrepancy argues against a generalized increase in thrombotic events associated with one treatment and raises the question of differences in PE detection.

Second, CTA utilization increased by approximately 50% between the two study periods. At the same time, the proportion of positive examinations increased from 2.4% to 9.0%. Increased diagnostic intensity may therefore have contributed to the higher observed PE incidence. The overlap of the second treatment period with the COVID-19 pandemic represents an additional potential temporal confounder.

Third, the chronological distribution of VTE events showed an episodic pattern rather than a distinct and sustained step change following the transition to tinzaparin. This temporal pattern does not provide clear support for a direct effect of the change in pharmacological agent.

Taken together, although both crude and adjusted analyses demonstrated an association between enoxaparin use and lower VTE odds in Group B, the additional analyses provide several plausible alternative explanations for this finding. The isolated increase in PE, changes in CTA utilization and diagnostic yield, and the absence of a clear temporal step change all argue against interpreting the observed association as definitive evidence of superior prophylactic efficacy of enoxaparin. The findings should therefore be considered hypothesis-generating and interpreted in light of the retrospective design, limited number of events, and potential residual and temporal confounding.

Apart from that, the inverse association between age and VTE observed in the multivariable analysis was an unexpected finding in our study. Although older age is generally considered a risk factor for VTE, older patients in our cohort had lower odds of VTE [3, 22]. This counterintuitive finding may partly reflect residual temporal confounding, as age differed between the treatment periods and is inherently correlated with the calendar period in this study. In addition, differences in time at risk and competing risks, particularly among older patients, may have contributed to the observed association.

### Comparison of rebleeding rates

In the second part of our study, we analysed the safety profiles of both agents by analyzing the incidence of postoperative rebleedings requiring surgical revision. We found no statistically significant differences in incidences of rebleedings between the two anticoagulants nor in the distribution of specific hematoma types, including spinal epidural, cranial epidural, subdural, and intracerebral hematomas. These findings suggest a comparable safety profile for both agents in the context of neurosurgical patients.

The overall rebleeding rates were low in both groups (1.36% vs. 1.42%) and showed a comparable incidence to previously reported studies in mixed neurosurgical patient populations, e.g. 0.6% in a study by Agnelli et al. in 2017 [1].

Comparison of our rebleeding rates with those reported in previous studies is challenging, as, to the best of our knowledge, only one study to date has directly assessed the efficacy and safety of tinzaparin versus enoxaparin in the immediate postoperative period [20]. However, given an expected rebleeding rate of approximately 1% and a sample size of around 500 patients, the study population is likely underpowered to detect a possible statistically significant difference in rebleeding rates between the two groups.

## Strengths and limitations

This study has several limitations. First, its retrospective design inherently restricts control over data completeness and introduces potential biases. Also, VTE events were assessed only during the inpatient stay; events occurring during rehabilitation or the post-discharge recovery phase at home were not captured, potentially leading to an underestimation of the true incidence of VTE. Apart from that, the change from enoxaparin to tinzaparin coincided with a change in calendar period, resulting in inherent collinearity between pharmacological agent and treatment period. Therefore, the independent effect of the pharmacological agent cannot be completely separated from temporal changes in clinical practice and other period-related factors. This is particularly relevant given the overlap of the tinzaparin period with the COVID-19 pandemic. Furthermore, the total number of VTE events was relatively small, limiting the statistical power and potentially reducing the stability of multivariable estimates. Lastly, the study population was heterogeneous, comprising patients with varying underlying pathologies and treatment regimens. While this heterogeneity may limit the comparability of subgroups, it reflects the clinical reality of most general neurosurgical centers. The relatively large overall sample size may, however, help mitigate the impact of individual variability.

## Conclusion

Both enoxaparin and tinzaparin were associated with low VTE rates and comparable postoperative rebleeding rates. Although higher VTE rates with tinzaparin were observed in the medium-to-high risk group, the predominance of PE, increased CTA utilization, and absence of a temporal step change limit a causal interpretation. These findings should therefore be considered hypothesis-generating rather than evidence of superior efficacy of either agent. Prospective studies are required to determine whether the observed difference is attributable to the pharmacological agent itself or primarily attributable to temporal and diagnostic factors.

## Data Availability

The datasets used and analysed during the current study are available from the corresponding author on reasonable request.
